# Innate Immune Responses in ALV-J Infected Chicks and Chickens with Hemangioma *In Vivo*

**DOI:** 10.3389/fmicb.2016.00786

**Published:** 2016-05-25

**Authors:** Min Feng, Manman Dai, Tingting Xie, Zhenhui Li, Meiqing Shi, Xiquan Zhang

**Affiliations:** ^1^Department of Animal Genetics, Breeding and Reproduction, College of Animal Science, South China Agricultural UniversityGuangzhou, China; ^2^Guangdong Provincial Key Lab of Agro-Animal Genomics and Molecular Breeding and Key Lab of Chicken Genetics, Breeding and Reproduction, Ministry of AgricultureGuangzhou, China; ^3^College of Veterinary Medicine, South China Agricultural UniversityGuangzhou, China; ^4^Division of Immunology, Virginia-Maryland Regional College of Veterinary Medicine, University of Maryland, College ParkMD, USA

**Keywords:** ALV-J, cytokines, innate immune response, chicks and chickens, *in vivo*

## Abstract

Avian leukosis virus subgroup J (ALV-J) infection can cause tumors and immunosuppression. Since the precise mechanism of the innate immune response induced by ALV-J is unknown, we investigated the antiviral innate immune responses induced by ALV-J in chicks and chickens that had developed tumors. Spleen levels of interleukin-6 (IL-6), IL-10, IL-1β, and interferon-β (IFN-β) were not significantly different between the infected chick groups and the control groups from 1 day post hatch to 7 days post hatch. However, IL-6, IL-1β, and IFN-β protein levels in the three clinical samples with hemangiomas were dramatically increased compared to the healthy samples. In addition, the anti-inflammatory cytokine IL-10 increased sharply in two of three clinical samples. We also found a more than 20-fold up-regulation of ISG12-1 mRNA at 1 day post infection (d.p.i.) and a twofold up-regulation of ZC3HAV1 mRNA at 4 d.p.i. However, there were no statistical differences in ISG12-1 and ZC3HAV1 mRNA expression levels in the tumorigenesis phase. ALV-J infection induced a significant increase of Toll-like receptor 7 (TLR-7) at 1 d.p.i. and dramatically increased the mRNA levels of melanoma differentiation-associated gene 5 (MDA5) in the tumorigenesis phase. Moreover, the protein levels of interferon regulatory factor 1 (IRF-1) and signal transducer and activator of transcription 1 (STAT1) were decreased in chickens with tumors. These results suggest that ALV-J was primarily recognized by chicken TLR7 and MDA5 at early and late *in vivo* infection stages, respectively. ALV-J strain SCAU-HN06 did not induce any significant antiviral innate immune response in 1 week old chicks. However, interferon-stimulated genes were not induced normally during the late phase of ALV-J infection due to a reduction of IRF1 and STAT1 expression.

## Introduction

Avian leukosis virus (ALV) is a member of the α-retrovirus genus of retroviridae, causing neoplastic disease, immunosuppression, and reproduction problems in the poultry industry worldwide. ALV strains are divided into 10 subgroups from A to J based on their viral envelope composition, host range, and cross-neutralization patterns (Payne et al., 1992; Barnard et al., 2006) avian leukosis> virus subgroup (ALV-J) isolates have been obtained from broiler breeders and laying chickens in most parts of China (Cui et al., 2003) and these infections result in serious economic losses in commercial layer flocks and local chicken breeds (Sun and Cui, 2007; Gao et al., 2010).

Avian leukosis virus subgroup J can induce the formation of different types of tumors such as haemangiomas and myelocytomas and immunosuppression due to ALV-J infection also increases susceptibility to other avian diseases (Abolnik and Wandrag, 2014). In the current study we analyzed the transcriptional level of selected immune-response genes we had previously identified from whole-transcriptome profiling of ALV-J-induced tumors in chicken spleen samples (Li et al., 2015). These genes included those with known anti-viral properties such as the single copy *Mx* gene, *ISG12-1* as well as the toll-like receptor *TLR-7* and other cytokine-related genes.

We investigated the effects of ALV-J infection on the *in vivo* mRNA and protein expression of related immune-response genes and cytokines during the early and late phases of infection. Our findings extend our current understanding of the host-response mechanism to exogenous ALV infection.

## Materials and Methods

### Ethics Statement

All animal research projects were sanctioned by the South China Agriculture University Institutional Animal Care and Use Committee. All animal procedures were performed according to the regulations and guidelines established by this committee and international standards for animal welfare.

### Virus

The ALV-J strain SCAU-HN06 (Zhang et al., 2011) was kindly provided by Dr. Weisheng Cao, South China Agricultural University. Infection with this virus leads to haemangioma development.

### Experimental Animals

Thirty-six 1-day-old specific-pathogen-free (SPF) White Leghorn chickens were hatched from eggs (Guangdong Da Hua Nong Animal Health Products Co., Ltd) and housed in isolator cages.

Three 140-day-old sick yellow chickens with neoplasms (designated SC1, SC2, and SC3) and three 140-day-old normal yellow chickens (designated NC1, NC2, NC3) were collected from the same flock from a farm in Guangdong Province, China.

### Sample Collection

A total of 36 1-day-old SPF chickens were randomly assigned to two groups: an SCAU-HN06-infected group and control group, each with eighteen chickens per group. Chickens were inoculated intraperitoneally at a dose of 0.2 mL (10^4.5^ TCID_50_/0.2 mL) of strain SCAU-HN06. The control group was injected with DMEM media alone. Spleens were aseptically collected from three infected chickens and three control chickens at 1, 2, 3, 4, 5, and 7 d.p.i., respectively.

Plasma samples were aseptically collected and centrifuged at 4°C at 2000 rpm for 12 min to isolate leukocytes. These were stored as viral stocks at -80°C. Spleens and livers from clinical samples were either stored at -80°C or fixed in 10% buffered formalin until use.

### Detection of SPF Chicken Infected with ALV-J

RNA was extracted from the spleens of SPF chickens (Fastagen, Shanghai, China) and reverse transcribed into cDNA (Thermo-Fisher Scientific, USA) using commercial kits. RT-PCR analyses was employed to detect ALV-J *via* specific primers (Smith et al., 1998).

### Clinical Sample Detection, Histopathology, and Immunohistochemical Staining

DNA was extracted from the spleens of the clinic samples using a commercial kit (Omega, USA). Specific primers were employed to differentiate between ALV-J, ALV-A/B, and other suspected viruses including Marek’s disease virus (MDV) and reticuloendotheliosis virus (REV) (Lai et al., 2011). According to a previously described method (Li et al., 2013), chicken plasma was used for virus isolation by inoculation into DF1 cells. DF-1 cells are only susceptible to exogenous ALV virions (Maas et al., 2006). Infected DF1 cell culture supernatants from clinical samples were tested for ALV group-specific antigen (p27) using the ALV Antigen Test Kit^R^ (IDEXX, USA) according to the manufacturer’s instructions. ALV-J infection was further confirmed by immunofluorescence using standard techniques (Venugopal et al., 1997). Infected cell images were collected using NIS-Elements BR analysis software (Nikon).

Liver tissue samples fixed in 10% buffered formalin were stained with hemotoxylin and eosin (HE) and examined histopathologically (Cheng et al., 2010). Immunohistochemical staining was used for further diagnoses with JE9 monoclonal antibody (kindly provided by Dr. Kun Qian, Yangzhou University). Binding of the JE9 antibody was detected using anti-mouse-HRP (Zhongshan Goldenbridge, Beijing, China).

### Analysis Expression of Related Gene of Innate Immunity by qRT-PCR

In our previous study, the transcriptome profiles of ALV-J-induced tumors in spleen samples compared to healthy spleen samples from White Recessive Plymouth Rock chickens were used to identify the genes related to ALV-J invasion (Li et al., 2015). In this study, the related genes of innate immune transcriptional responses were analyzed according to the transcriptome profiles.

Expression of related genes of innate immunity were analyzed by quantitative real-time polymerase chain reaction (qRT-PCR). Total RNA was extracted from frozen spleens of SPF chickens and clinical samples using the RNAfast200 kit (Fastagen), followed by cDNA synthesis of mRNA with the RevertAid First strand cDNA synthesis kit (Thermo-Fisher Scientific) according to the manufacturer’s instructions. qRT-PCR was performed on a Biorad CFX96 Real-Time Detection System using iTaq^TM^ Universal SYBR^®^ Green Supermix Kit reagents (Biorad, CA, USA) according to the manufacturer’s specifications. Primers used for qRT-PCR were designed using the NCBI Primer BLAST program^1^ and were based on published target sequences (**Table 1**). Data analyses were performed using the 2^-ΔΔCt^ method (Livak and Schmittgen, 2001).

**Table 1:** qRT-PCR primers.

Target	Primer	Sequence (5′-3′)	Accession no.	Reference
GAPDH	Forward	GAACATCATCCCAGCGTCCA	NM_204305.1	Dai et al., 2015
	Reverse	CGGCAGGTCAGGTCAACAAC		
ISG12-1	Forward	TAAGGGATGGATGGCGAAG	NM_001002856	Kint et al., 2015
	Reverse	GCAGTATCTTTATTGTTCTCAC		
ZC3HAV1	Forward	TTGATTCGGCGCCTCTCTAC	NM_001012938.1	
	Reverse	ACTGGCCGTGGTCATTCTTC		
TLR3	Forward	GGTCCAGCTTTCAAGAGCCT	NM_001011691.3	
	Reverse	GCAACACCAGAGTACCGTGA		
TLR7	Forward	TCTGGACTTCTCTAACAACA	NM_001011688.2	Zhou et al., 2013
	Reverse	AATCTCATTCTCATTCATCATCA		
TLR4	Forward	AGGCACCTGAGCTTTTCCTC	NM_001030693.1	
	Reverse	TACCAACGTGAGGTTGAGCC		
MDA5	Forward	ATTCCACAGCCGCAGATTC	NM_001193638.1	Hayashi et al., 2014
	Reverse	CAAGATTGGCACAGATTTTCAGA		

### Measurement of Cytokines by ELISA

Spleen homogenates of SPF and clinical chicken samples were used to detect cytokine protein levels. Spleen tissues were rinsed in ice-cold PBS to remove excess blood thoroughly and weighed before homogenization. Homogenized them in 1mL of PBS with a steel ball using TissueLyser II (QIAGEN, Germany). The resulting suspension was subjected to two freeze-thaw cycles to further break the cell membranes. The homogenates were centrifugated for 5 min at 5000 × *g* and the supernatant was removed and was assayed immediately. Enzyme-linked immunosorbent assay (ELISA) kits for chicken interferon-β (IFN-β), interleukin-1β (IL-1β), IL-6, and IL-10 determination were obtained from Wuhan USCN (Cloud-Clone Corp. China). The ELISA experiments were performed according to the manufacturer’s specifications.

### Western Blot Analysis

Tissue homogenates were prepared from spleens of chickens with tumors and normal chickens as above described. The lysates were collected and incubated on ice for 10 min and then cleared by centrifugation at 10,000 *g* for 5 min at 4°C. Total protein content was determined with a BCA protein assay kit (Life Technologies, USA). Total protein (20 μg) was resolved by 12% SDS-PAGE and transferred onto nitrocellulose membranes (Whatman, Maidstone, UK). Membranes were blocked with 5% w/v skim milk for 2 h at 37°C, and then incubated overnight at 4°C with mouse anti-GAPDH antibody (Beyotime Inst Biotech, Shanghai, China), rabbit anti-interferon regulatory factor 1 (IRF-1) and rabbit anti-signal transducer and activator of transcription 1 (STAT1) antibodies (LSBio, Seattle, WA, USA). After three rinses with PBS Tween 20 (PBST) buffer, the membranes were incubated at 37°C for 1 h with anti-rabbit-HRP or anti mouse-HRP (Zhongshan Goldenbridge, Beijing, China) that had been diluted in PBST. Membranes were washed three times with PBST and signals were detected using an ECL kit (Zhongshan Goldenbridge, Beijing, China).

### Statistical Analyses

Statistical comparisons were made by GraphPad Prism 5 (GraphPad Software Inc., San Diego, CA, USA) and statistical significance was represented by *P* values of >0.05, <0.05,0.01, or 0.001.

## Results

### Detection of Samples

**FIGURE 1:**
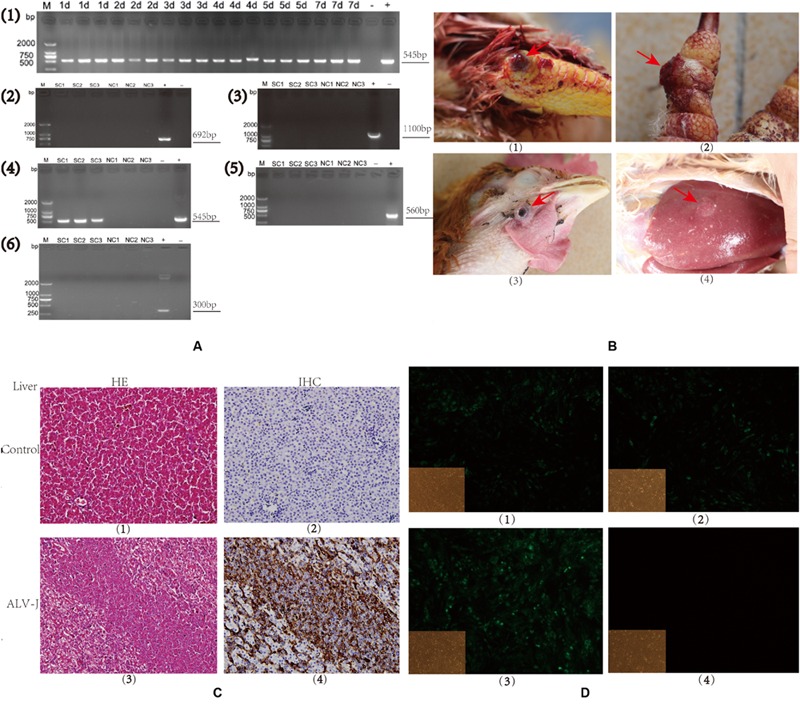
**Detection of avian leukosis virus subgroup J (ALV-J) in specific-pathogen-free (SPF) chicks and clinical samples. (A)**. Agarose gels of RT-PCR and PCR products from chicken samples using virus-specific primer sets. (1). Time course of an SCAU-HN06 infection in SPF chicks using primers H5/H7 by RT-PCR. (2–6). PCR results using primers specific for (2). ALV-A, (3). ALV-B, (4). ALV-J, (5). MDV, and (6). REV in clinical samples. Chicken sample names are listed above each gel lane; (–) negative control, (+), positive control, bp = base pairs. Numbers on the left indicate lengths of molecular weight standards. **(B)**. Photographs of tumors (arrows) from infected (SC group) chickens: (1). Joint hemangioma, SC1 (2). Digit hemangioma, SC2; (3) Neck hemangioma, SC3 (4) Liver tumor, SC3. **(C)**. Liver tissues from an uninfected (NC3) and an ALV-J-infected chicken (SC3; 400 × magnification). (Panels 1 and 3) Hemotoxylin and eosin (HE) staining; (Panels 2 and 4) Immunohistochemical staining for virus-specific protein gp85 using monoclonal antibody JE9. **(D)**. Immunofluorescence of DF-1 cells using an ALV-J-specific antibody JE9 (150 × magnification). (1, 2, and 3) Cells infected using virions isolated from chickens SC1, SC2, and SC3; (4) Uninfected DF-1 cells as negative control.

To determine whether the SPF chickens were successfully infected, we measured gene expression levels of ALV-J-specific genes from chickens infected with the SCAU-HN06 virus strain. RT-PCR tests of spleen samples from SPF chickens at 1–7 d.p.i. using ALV-J-specific primers were all positive (**Figure 1A**, Panel 1). ALV-J could not be detected in the control animals (data not shown).

To verify that the clinical tumors were induced by ALV-J and no other oncogenic viruses, we used additional methods for pathogen detection. The presence of hemangiomas is a characteristic of ALV-J infection. These tumors were evident on the skin of the joints and digits, in the ocular region and in the livers of birds from the same flock (**Figure 1B**, Panels 1–4).

Histologically, the tumors were typical cavernous hemangiomas with malignant vessel hyperplasia visible as a closely packed meshwork of blood vessels. Microscopically, the tumor cells were relatively uniform large myeloid cells and lymphoid cell hyperplasia was observed in liver (**Figure 1C**, Panel 3). Bluish blisters were also observed and some of these lesions were associated with continuous bleeding.

We also observed a significant up-regulation of viral gp85 expression in tumor cells from ALV-J-infected livers using immunohistochemical staining with the JE9 monoclonal antibody (**Figure 1C**, Panel 4). PCR tests on the genomic DNA of the three sick chicken spleens using ALV-J-specific primers were all positive (**Figure 1A**, Panel 4). We detected no related viral infections as judged by the absence of amplicons using primers specific to ALV-A, ALV-B, MDV, and REV (**Figure 1A**, Panels 2, 3, 5, 6). The PCR tests of three normal chickens from the same flock were negative using all the listed primers (**Figure 1A**, Panels 2, 3, 5, 6).

We could also identify the presence of virion-encoded p27 from the sick, but not the healthy chickens (data not shown). Furthermore, immunofluorescence of infected cells using an ALV-J-specific monoclonal antibody were all positive (**Figure 1D**, Panels 1–3) and the uninfected control completely lacked any signal (**Figure 1D**, Panel 4). These results confirmed the presence of ALV-J in the clinical samples as infections caused by a single pathogen.

### Differential Expression of the Innate Immune Genes in the Early and Late Phases of ALV-J Infection

**FIGURE 2:**
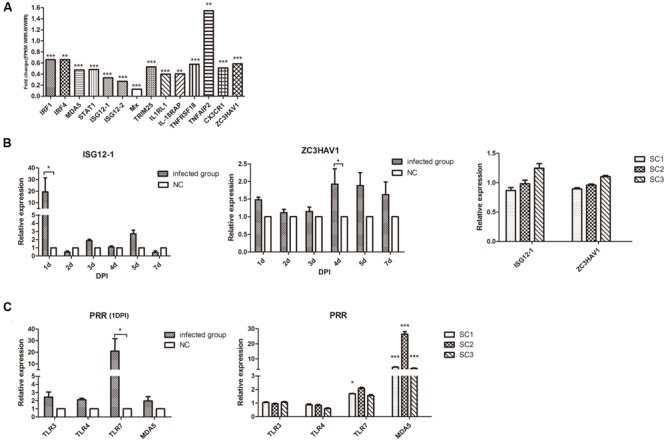
**Transcriptional analysis of IFN-stimulated and pattern recognition receptor (PRR) genes in chicks and chickens with tumors. (A)**. transcriptome analysis of splenic genes related to innate immunity based on our previous study of ALV-J-induced tumors in White (Recessive) Plymouth Rock chickens (Li et al., 2015). **(B)** Quantitative analysis of the expression of *ISG12-1* and *ZC3HAV1* in chicks (Left) and chickens with tumors (Right). **(C)** Quantitative analysis of the expression of PRRs in chicks (Left) and chickens with tumors (Right). Expression is relative to the average of the NC group (*n* = 3) for **(B)** Right and **(C)** Right. ^∗^*p* < 0.05, ^∗∗^*p* < 0.01, ^∗∗∗^*p* < 0.001.

According to the transcriptome profiles of ALV-J-induced tumor spleen samples and healthy spleen samples from White (Recessive) Plymouth Rock chickens in our previous experiments (Li et al., 2015), we analyzed the transcriptional level of related innate immune genes. As a guide, we compared healthy spleens to ALV-J-infected spleen samples that possessed tumors and found a general decreasing trend except for TNFAIP2 which increased slightly (**Figure 2A**). Genes that were decreased two to eightfold included interferon-stimulated genes such as the single copy antiviral gene *Mx*, IFNα-stimulated genes 1 and 2 (*ISG12-1, ISG12-2*) and *ZC3HAV1* (zinc finger CCCH-type antiviral protein 1) (**Figure 2A**). Considering this data, we examined whether ALV-J induces or inhibits innate immune host responses during both early and late phases of infection.

Avian leukosis virus subgroup J infection in the early stages resulted in a more than 20-fold up-regulation of ISG121 mRNA at 1 d.p.i. (*p* < 0.05) and greater than twofold up-regulation of ZC3HAV1 mRNA at 4 d.p.i. (*p* < 0.05). However, ALV-J late infection exhibited no statistical differences in ISG12-1 and ZC3HAV1 mRNA expression in the tumorigenesis phase (*p* > 0.05) (**Figure 2B**).

Avian leukosis virus subgroup J also induced a significant increase of TLR-7 at 1 d.p.i. (*p* < 0.05) as well as melanoma differentiation-associated gene 5 (MDA5) in the tumorigenesis phase (*p* < 0.001) (**Figure 2C**). These results suggest that ALV-J was primarily recognized by chicken TLR7 and induced *ISG12-1* expression at 1 d.p.i. Chicken MDA5 was the main ALV-J-sensing pattern-recognition receptor during the late infection phase *in vivo*.

### Differential Expression of Cytokines in the Early and Late Phases of ALV-J Infection

**FIGURE 3:**
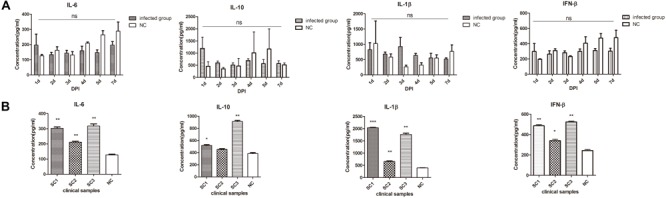
**Differential expression of cytokines in the early and late phases of ALV-J infection. (A)** IL-6, IL-10, IL-1β, and IFN-β cytokine expression measured by ELISA between the infected chick group and the control group (NC). **(B)**. Cytokine expression measured by ELISA in chickens with neoplasms (SC1–3) and uninfected control (average of NC group, *n* = 3). ^∗^*p* < 0.05, ^∗∗^*p* < 0.01, ^∗∗∗^*p* < 0.001.

To further explore the differences on innate immune responses between the early and late phases of ALV-J infection, we measured cytokine levels in spleen homogenates comparing healthy chickens to chickens with ALV-J infection. Interestingly, IL-6, IL-10, IL-1β, and IFN-β showed no significant differences between the groups of SPF chicks from 1 to 7 d.p.i. (*p* > 0.05) (**Figure 3A**). However, clinical chickens with neoplasms showed IL-6, IL-1β and IFN-β levels that were significantly altered within the clinical group (**Figure 3B**). In addition, the anti-inflammatory cytokine IL-10 was also increased sharply in two of three clinical samples with neoplasms (**Figure 3B**).

Taken together these results indicated that ALV-J early infection induced no obvious antiviral innate immunity responses in chicks sampled from 1 to 7 d.p.i. However, this was not the case for late infections and there were significant increases in Type I IFN, pro-inflammatory cytokines as well as IL-10.

### Western Blot Analysis of the IRF1 and STAT1 Expression in the Late Phases of ALV-J Infection

**FIGURE 4:**
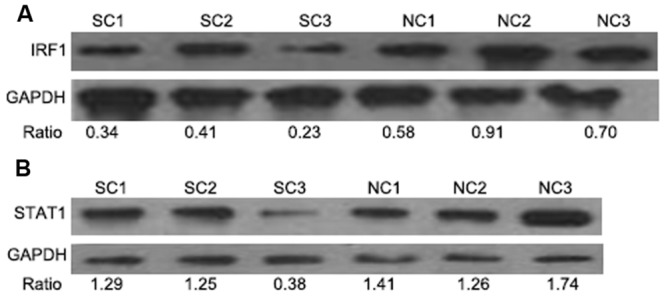
**Western blot analysis of IRF1 and STAT1 levels in the late phases of ALV-J infection.** IRF1 and STAT1 expression in the spleens of chickens with neoplasms (SC1–3) compared to healthy controls (NC). **(A,B)** represent the detection of IRF1 and STAT1 using WB, respectively.

The JAK-STAT pathway as well as IRF-1 are key regulators of viral immune responses. Therefore we measured expression levels of IRF1 and STAT1 *via* Western blotting. Compared with the control uninfected chickens, IRF1 and STAT1 levels were decreased in the tumor samples. This was especially true with chicken SC3 that also displayed liver tumors (**Figures 4A,B** and see **Figure 2A**).

## Discussion

Innate immunity plays a dominant role in antiviral responses of chickens at 1–5 days of age due to the incomplete structural organization of their secondary immune organs (Mast and Goddeeris, 1999). ALV transmission primarily occurs at hatching or in the 1st weeks of life (Witter and Fadly, 2001). Accordingly, we deliberately studied the innate immune response of chicks within a week of hatching in response to ALV-J infection. Our findings showed that cytokine levels were not significantly different between the infected chick group and the control group from 1 to 7 d.p.i. (*p* > 0.05). Even so, IL-6, IL-10, and IFN-β levels were all increased immediately after infection at 1 d.p.i. (**Figure 3A**). It was also at this time-point that *ISG12-1* and *TLR7* expression levels were dramatically increased. Hence, day 1 of infection plays a pivotal role in innate immune responses of chicks to some degree.

Retroviruses can selectively trigger an array of innate immune responses through various pattern recognition receptors (PRRs) (van Montfoort et al., 2014). However, the nature of the exact innate sensors that detect ALV-J had remained elusive until now. Recognition of HIV-1 by TLR7 does not require retroviral replication, and only requires attachment and endocytosis (van Montfoort et al., 2014). Our results showed that ALV-J induced a significant increase of TLR-7 at 1 d.p.i., so we speculated that ALV-J was primarily recognized by chicken TLR7 at 1 d.p.i. The cytoplasmic sensor RIG-I can serve as a sensor for HIV genomic RNA (van Montfoort et al., 2014) and Chickens lack RIG-I, but the MDA5 can partially compensate to generate an interferon response (Magor et al., 2013). As host mRNAs, retroviral genomic RNAs are capped and polyadenylated (Solis et al., 2011). During the tumor phase *in vivo*, ALV-J had been integrated into the host genome (Li et al., 2014). ALV-J induced a significant increase of mRNA expression of MDA5 in the tumorigenesis phase (*p* < 0.001), we speculated that MDA5 was the main sensing receptor during the late phase *in vivo* and this is consistent with previous reports (Hang et al., 2014).

Since IL-6 and IFN-β can induce the expression of IFN-stimulated genes by activating the JAK-STAT pathway (Hoffmann et al., 2015; Wang and Zhang, 2015), we speculated that ALV-J was primarily recognized by chicken TLR7. This would lead to regulation of ISG12-1 expression *via* JAK-STAT pathway activation at 1 d.p.i. However, taken together, the early antiviral innate immune response was too weak to resist ALV-J invasion as evidenced by the lack of obvious cytokine expression. In fact, the SPF chicks were susceptible to ALV-J within 1–7 days post hatch.

During late infection stages, the secretion levels of IL-6, IL-1β, and IFN-β in the three clinical samples with neoplasms had significantly increased. Of note was the anti-inflammatory cytokine (IL-10) that possessed immunosuppressive effects (Sabat et al., 2010), and this cytokine was sharply increased in the two of three clinical samples. In other words, ALV-J late infection caused significant immune responses including increasing type I IFN, pro-inflammatory as well as anti-inflammatory cytokines. However, there were no statistical differences in ISG12-1 and ZC3HAV1 mRNA expression in the tumorigenesis phase. We speculated that functional ISGs cannot be induced in the late infection phase.

As a tumor suppressor, *IRF1* expression is decreased in a variety of human cancers (Connett et al., 2005; Wang et al., 2007). IRF1 can also exert antiviral activity by inducing interferon-stimulated gene expression directly (Stirnweiss et al., 2010). Our previous study demonstrated that *IRF1* expression was decreased as a target of miR-23b in the White (Recessive) Plymouth Rock chickens with ALV-J infection (Li et al., 2015). In that study we also showed that the antiviral activity of IRF1 was inhibited during the late phase of ALV-J infection.

There are a number of reports concerning JAK-STAT pathway inhibition caused by reducing *STAT1* expression or by inhibiting its phosphorylation (Precious et al., 2005; Audsley and Moseley, 2013). Our previous transcriptome results and the Western blotting data presented here demonstrate that STAT1 levels are decreased in chickens with tumors induced by ALV-J infection. We hypothesize that ALV-J escapes through inhibition of the host antiviral immune response by modulating the JAK-STAT signaling pathway. Additional studies concerning this hypothesis are currently being conducted.

In summary, the present study demonstrates that the ALV-J strain SCAU-HN06 produces an almost undetectable antiviral innate immune response in 1 week old chicks. Cytokines were induced in the yellow chickens with tumors caused by ALV-J infection. However, IFN-stimulated gene expression was not induced normally during the late phase of ALV-J infection. This is most likely the result of a reduction in *IRF1* and *STAT1* expression.

## Author Contributions

MF participated in the design of the study, performed the experiments, collected and analyzed data, and drafted the manuscript. MD performed western blot assay. TX and ZL helped with the animal experiment. MS and XZ participated in the design and coordination of the study. All authors read and approved the final manuscript.

## Conflict of Interest Statement

The authors declare that the research was conducted in the absence of any commercial or financial relationships that could be construed as a potential conflict of interest.

## Footnotes

1 http://www.ncbi.nlm.nih.gov/tools/primer-blast/
